# Phosphorylation Patterns of Preribosomal Proteins Associated with the RNA Exosome

**DOI:** 10.1021/acs.jproteome.6c00095

**Published:** 2026-06-29

**Authors:** Felipe A. Almeida, Valdir Gomes Neto, Rebeca B. Jantsch, Moises R. A. Barros, L. Paola P. Cepeda, Bruno R. S. Queiroz, Anna B. Machado, Ana P. J. Menezes, Julia P. C. Da Cunha, Carla C. Oliveira

**Affiliations:** † Department of Biochemistry, Institute of Chemistry, 28133 University of São Paulo, São Paulo, São Paulo 05508-000, Brazil; ‡ Cell Cycle Laboratory, Center of Toxins, Immune Response and Cell Signaling-Center for Research on Toxins, Immune-Response, and Cell Signaling (CeTICS), 196591 Butantan Institute, São Paulo, São Paulo 05503-900, Brazil

**Keywords:** RNA exosome, rRNA processing, ribosome biogenesis; affinity purification, proteomic analysis, protein phosphorylation, protein interactions, nucleolar proteins, protein-RNA interactions, *Saccharomyces cerevisiae*

## Abstract

The RNA exosome is an essential and ubiquitous RNase with exonucleolytic activity that is involved in ribosome biogenesis and RNA quality control in eukaryotes. It is present both in the nucleus and cytoplasm and interacts with specific cofactors in each cell compartment, which are essential for the recruitment and activity control of the exosome. Post-translational modifications are known to regulate enzyme activity and protein interaction, although their precise roles are individually specific. In this study, we investigated the phosphorylation status of proteins associated with the nuclear (Rrp6) and core (Rrp46) subunits of the RNA exosome in *Saccharomyces cerevisiae.* Using coimmunoprecipitation followed by phosphopeptide enrichment and high-resolution mass spectrometry, we identified 114 phosphorylation sites on proteins functionally related to rRNA processing. Differential phosphorylation patterns between Rrp6 and Rrp46 coimmunoprecipitations are consistent with distinct exosome assemblies and suggest potential regulatory roles for phosphorylation. Several phosphosites were identified in exosome subunits and cofactors, revealing potential regulatory mechanisms for fine-tuning exosome function. The results shown here highlight the role of phosphorylation in the recruitment and control of the exosome in RNA processing and degradation, offering new insights into the post-transcriptional control of gene expression.

## Graphical abstract

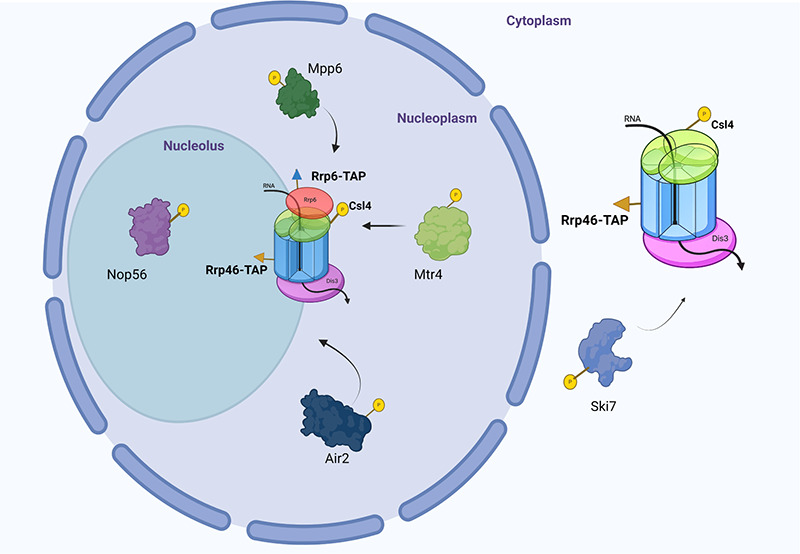

## Introduction

The RNA exosome complex is a ubiquitous RNase with 3′-5′ exonucleolytic activity involved in processing and degradation of all classes of RNA in eukaryotes.^1,2^ In budding yeast, the exosome is comprised of 11 different protein subunits, nine of which form the catalytically inactive core (Csl4, Rrp4, Rrp40–43, Rrp45, Rrp46, Mtr3). The core associates with Rrp44/Dis3 in the cytoplasm, forming Exo-10, and in the nucleus, Exo-11 is composed of Exo-10+Rrp6. Additionally, the exosome binds to specific cofactors in each subcellular compartment.^3^ The exosome was first identified in the yeast *Saccharomyces cerevisiae* and later detected in other eukaryotes and some archaea species.^4–6^ In the nucleus, the exosome plays an essential role in pre-rRNA processing and snoRNA and snRNA maturation.^7^

The exosome is recruited to the 90S SSU processome, the first preribosomal particle to be formed cotranscriptionally, and is responsible for the degradation of the 5́'-ETS spacer sequence after its release from the 35S pre-rRNA.^8,9^ Later during ribosomal maturation, the exosome is recruited to the pre-60S particles to process the 7S intermediate and form the mature 5.8S rRNA.^7,10–12^

The major interactors of the exosome in the nucleus are responsible for its recruitment to preribosomal particles during maturation and include the RNA helicase Mtr4, which is also a subunit of the TRAMP complex, and ribosome assembly factors Nop53 and Utp18, which recruit the exosome through Mtr4.^8,12–15^ In the cytoplasm, the exosome is recruited by the SKI complex to degrade its substrates.^16,17^ Ski7 is a critical adaptor connecting the SKI complex (Ski2-Ski3-Ski8) and the cytoplasmic exosome for mRNA degradation.^18^

Although the structure and function of the exosome have been extensively studied in recent years, a detailed understanding of how the control of its function and recruitment is achieved is still lacking. Post-translational modifications are a very important mechanism for controlling protein function, and protein phosphorylation is one of the most prevalent modifications. Phosphorylation of ribosomal proteins has long been known to influence translation^19–21^ and has also been shown to affect ribosomal maturation and interaction between assembly factors.^22–24^ Phosphorylation at specific sites on Dis3 (Ser809 and Tyr814) and Mtr4 (Thr1061 and Ser1067) from *Schizosaccharomyces pombe* has been shown to regulate exosome function.^25^ In this study, we investigated the role of phosphorylation in the regulation of the *Saccharomyces cerevisiae* exosome interaction with its cofactors. We selected Rrp6 and Rrp46 as representative components of the nuclear and core exosome complexes, respectively, to identify phosphorylation sites of proteins interacting with exosome-containing complexes that may be regulated during ribosome biogenesis or cytoplasmic RNA processing/degradation processes.

## Materials and Methods

### Yeast Strains and Culture Conditions

Exosome subunits were affinity-purified from strains RRP46-TAP::HIS3 and RRP6-TAP::URA3 (Euroscarf) as described.^26^ Maintenance and growth of yeast cultures were performed according to standard procedures (Sherman et al., 1986). They were grown at 30 °C in minimal medium (YNB) supplemented with 2% glucose, in addition to the essential amino acids, according to their auxotrophic markers. The yeast wild-type strain W303 was used as a negative control for subsequent analysis.

### Cloning and Site-Directed Mutagenesis

For cloning of *RRP4* and *SKI7*, the plasmids were linearized by inverse PCR, and the inserts were amplified from the yeast genomic DNA and recombinant plasmids were constructed with the InFusion HD (Clontech) recombination cloning system using competent Stellar (Clontech) cells. Site-directed mutagenesis was performed by overlap of PCR products and the InFusion DNA assembly kit, following the manufacturer’s instructions.

### Spot Assay Growth and Growth Curve

Precultures of the conditional strain *Δski7/yCP111-MET15:SKI7-TAP* ^27^ and *GAL1:13MYC-RRP4/yCP111-ADH1:RRP4–3HA*, expressing the corresponding plasmid-borne wild-type Ski7 or Rrp4, or mutants (Ski7-S90A and Rrp4-S152A) in log phase, were diluted for OD 600 nm = 0.1 and then subjected to 10-fold serial dilutions, and spotted onto glucose-containing solid minimal medium, methionine-free or methionine-containing (2 mM) and incubated at 30 °C. Growth curve measurements were performed in microplates incubated over 12 h of growth using a BioTek LogPhase 600 Microbiology Reader set to read OD600 every 60 min at 30 °C in the case of Ski7, and at 30 and 37 °C in the case of Rrp4.

### Coimmunoprecipitation of Rrp46, Rrp6, and W303 Strains

For the tandem affinity purification (TAP) method, these yeast strains were cultured in 6L YPD (1% yeast extract; 2% peptone; 2% glucose) medium. Biological triplicates were harvested at log phase (OD_600_ ∼ 1) by centrifugation at 8000 rpm (F12–6 × 500 LEX Rotor, Sorvall R6 C Plus) for 30 min at 4 °C. The cell pellets were resuspended in cooled extraction buffer (60 mM Tris–HCl pH 8.0, 50 mM NaCl, 10 mM MgCl_2_, 5% glycerol, 1% PMSF, 1% triton, 1% EDTA, Pierce Protease Inhibitor Tablets, and Pierce Phosphatase Inhibitor Tablet) and flash-frozen in liquid N2.

The coimmunoprecipitation assays were carried out as previously described,^28^ with minor modifications. Briefly, the whole-cell lysates were obtained by cryogenic milling on a Ball Mill device (Retsch PM 100) and cleared by ultracentrifugation at 28,100 rpm (P50AT4 rotor, CP80NX Hitachi) for 60 min at 4 °C. The lysates were incubated for 2 h at 4 °C with 200 μL IgG-Sepharose 6 Fast Flow (GE Healthcare) previously equilibrated with extraction buffer, and centrifuged at 500 rpm at 4 °C for 2 min. The beads were washed twice with extraction buffer and twice with wash buffer (50 mM tris-HCl and 50 mM NaCl). Elution was performed in the same column by adding 1 mL of TEV cleavage buffer and 100 units of TEV protease. The beads were rotated overnight at 4 °C, and the eluate was recovered by gravity flow. Eluates were lyophilized and stored at −80 °C until treatment for LC–MS/MS analysis. Both input and eluate fractions were separated for analysis by SDS-PAGE and Western blot.

### SDS-PAGE and Immunoblot

Aliquots (∼5%) of yeast coimmunoprecipitated proteins were resuspended in SDS sample buffer, resolved on 10% SDS-PAGE (Tris-glycine running buffer), transferred to nitrocellulose membranes (Ambion), and incubated with an antibody against the CBP tag (Millipore). Secondary antibody conjugated to IRDye680RD (antirabbit IgG, LI-COR) was employed, and near-infrared Western blot detection was carried out using ChemiDoc MP Imaging System (Bio-Rad).

### Peptide Digestion and TiO_2_ Phosphopeptide Enrichment

The lyophilized eluates were subjected to in-solution digestion for LC–MS/MS analysis. Briefly, proteins were resuspended in 50 mM NH_4_HCO_3_, 8 M urea, and reduced with DTT at a final concentration of 5 mM at 56 °C for 25 min. Samples were then alkylated with 14 mM iodoacetamide for 30 min under protection from light. After dilution with 50 mM NH_4_HCO_3_, samples were digested for 16 h with sequencing-grade modified trypsin (Promega) at a 1:50 (E:S) ratio at 37 °C. The proteolysis was stopped by adding TFA to a final concentration of 0.4% (v/v), and the resultant peptides were desalted using SepPak tC18 cartridges (Waters).

A High-Select TiO_2_ kit (Thermo Fisher Scientific) was used to enrich phosphopeptides from tryptic peptides. The digested peptides were placed into TiO_2_ spin tips, and the enrichment steps were performed according to the manufacturer’s instructions. After elution of the phosphopeptides, the eluate was vacuum-dried and stored at −80 °C until mass spectrometry analysis.

### Mass Spectrometry Analysis and Data Acquisition

Digested peptides were resuspended in 0.1% formic acid (FA) and separated on an in-house reversed-phase capillary emitter column (10 cm × 75 μm, filled with 5 μm particle diameter C18 Aqua resins-Phenomenex) coupled to a nano-HPLC (Thermo). Mobile phases consisted of 0.1% FA in water (buffer A) and 0.1% FA in acetonitrile (buffer B). Peptides were eluted at 300 nL min^–1^ using a 60-min gradient (2–28% B for 45 min, 28–80% B for 13 min, and a return to 5% B in 2 min). The eluted peptides were analyzed by an LTQ-Orbitrap Velos (Thermo Scientific) (source voltage of 1.9 kV, capillary temperature of 200 °C). The mass spectrometer was operated in DDA mode with dynamic exclusion enabled (exclusion duration of 45 s), MS1 resolution of 30,000, and MS2 normalized collision energy of 30. For each cycle, one full MS1 scan range of 200–2000 *m*/*z* was followed by ten MS2 scans (for the most intense ions) using an isolation window size of 1.0 *m*/*z* and collision-induced dissociation (CID). The 10 most intense precursor ions were selected for fragmentation by CID in DDA. The raw data from the LTQ Velos-Orbitrap were analyzed with the MaxQuant (version 2.6.7.0) software using the UniProt database for *Saccharomyces cerevisiae* (strain ATCC 204508/S288c), setting tolerance for the MS of 20 ppm and the MS/MS of 0.5 Da; carbamidomethylation of cysteine as a fixed modification; phosphorylation at STY residues as variable modifications; and 1% false discovery rate (FDR). The match-between-runs option was used to increase the number of trusted IDs. The normalized intensity values of the Phospho (STY) Sites.txt output and the intensity columns for each biological replicate were used for downstream analyses. Identifier for the raw mass spectrometry data: PXD073587, available via PRIDE.

### Label-Free Phosphoproteomics Data Analysis

Initial data analysis was performed using Perseus software (version 2.1.3.0, Max Planck Institute of Biochemistry). The affinity purification assays using different baits were analyzed together. The MaxQuant output data was initially filtered by removing contaminants, hits to the reverse database, and phosphoproteins with localization probability ≤ 0.70. The intensities were log2-transformed, and the biological triplicates were grouped by categorical annotation in the two conditions assessed, Rrp46 and Rrp6. Furthermore, phosphopeptides were filtered to include only those detected in at least two of the three biological replicates or present uniquely in one condition (presence/absence filtering for exclusive identifications).

For differential phosphorylation analysis, intensity values log_2_-transformed were subjected to Student’s *t*-test. Only phosphopeptides with statistically significant differences (FDR-adjusted p-value (Benjamini–Hochberg) ≤ 0.05) were considered for downstream analysis. Additionally, phosphopeptides identified in control W303 samples were excluded to reduce false-positive identifications.

### Bioinformatic Analysis

To refine the phosphoproteomic data set toward exosome-related processing, the identified phosphoproteins were filtered using the Perseus annotation tool. Proteins were manually inspected and classified into two categories: (i) candidates involved in rRNA processing and nucleo-cytoplasmic transport, and (ii) all remaining proteins. This classification is provided in Supplementary Table 1 (Suppl. Table 1), where the column “BP” assigns 1 to proteins not associated with these pathways and values 2–10 to proteins grouped into specific rRNA-processing and transport subprocesses. Only the curated candidates (BP = 2–10) were used as input for the subsequent analysis. The heatmap was generated from the curated list of candidate phosphosites filtered in Perseus, using their log_2_-transformed phosphorylation intensities. Each row represents a phosphosite, and each column corresponds to a biological replicate within each condition. The processed matrix was imported into Python, alphabetically ordered by phosphosite, and samples were grouped by condition to support direct comparison of phosphorylation patterns. The color bar was derived from the full range of log_2_ values in the data set. This scale reflects the relative abundance of each phosphosite, with higher intensities indicating more abundant phosphosites and lower values representing reduced signals.

The biological process and molecular function were retrieved directly from the Gene Ontology resource and subsequently consolidated into higher-order categories for interpretation. To contextualize phosphosites structurally, selected residues were mapped onto crystallographic models of exosome-associated cofactors using PDB entries 6FSZ (Rrp4) and 5GO6 (Ski7). Structural visualization using Chimera v. 1.19 software allowed assessment of whether each phosphosite lies at RNA-binding surfaces, protein–protein interaction interfaces, or flexible regions, complementing the GO-based functional assignments. Phosphosite conservation was assessed across eukaryotes using an automated workflow that combines BLAST-based ortholog detection with alignment-guided residue mapping. Curated *S. cerevisiae* phosphosites (UniProt ID, modified residue, and position) were standardized into unique identifiers and used to retrieve the corresponding full-length protein sequences. These sequences were submitted to BLASTp searches against a curated multispecies proteome database, and putative orthologues were accepted only when supported by reciprocal best hits. Each orthologue set was then aligned with MAFFT (L-INS-i), and the *S. cerevisiae* phosphosite coordinates were projected onto the alignments to extract the homologous amino acid in each species. This procedure yielded a comprehensive site call table capturing both the exact residues and their biochemical class (STY, acidic DE, other residues, or gaps).

## Results

To investigate the possible regulatory role of phosphorylation in the recruitment of the exosome to preribosomal complexes, we conducted a label-free phosphoproteomic analysis using *Saccharomyces cerevisiae* strains expressing TAP-tagged versions of the nuclear (Rrp6) or core (cytoplasmic/nuclear—Rrp46) subunits of the RNA exosome. These subunits interact with distinct protein partners and ribonucleoprotein complexes during ribosome biogenesis or cytoplasmic RNA degradation, and our goal was to identify phosphorylation events that may modulate these interactions. Protein coimmunoprecipitation experiments were therefore performed using total extracts of yeast cells expressing either Rrp6-TAP or Rrp46-TAP. Eluted proteins from biological triplicates were then digested with trypsin, and the resulting peptides were subjected to phosphopeptide enrichment and identification by mass spectrometry. The protein baits were detected both by mass spectrometry and immunoblots (Figure 1A). Extracts from wild-type W303 strain, processed in parallel, were used, and proteins/phosphosites detected in control runs were excluded from downstream analyses to reduce background binders. The similarity between replicates was assessed using principal component analysis (PCA), which revealed two well-defined clusters, corresponding to the Rrp46 and Rrp6 samples, with clear separation between conditions and tight grouping of the biological replicates within each cluster (Figure 1B).

**Figure 1:**
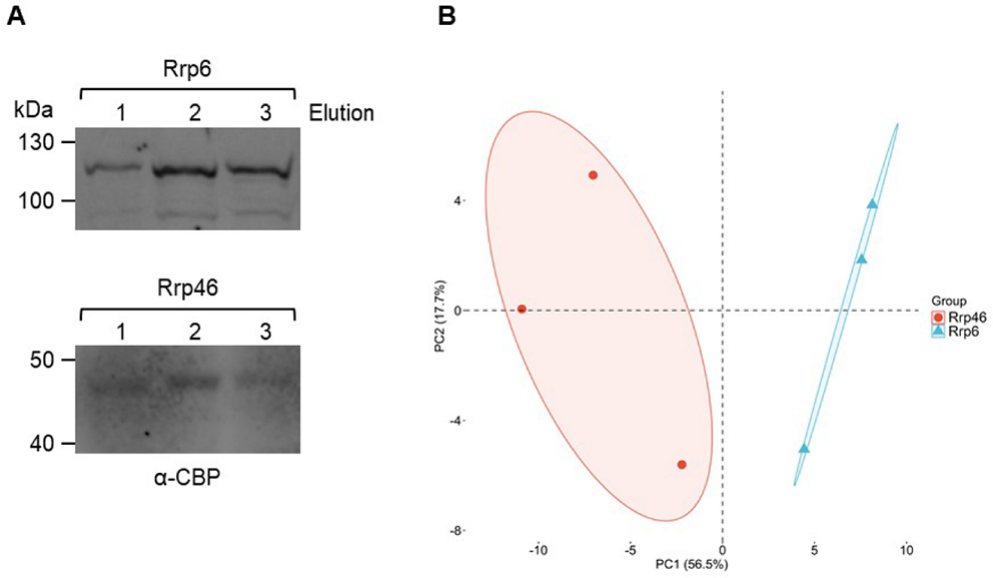
Protein coimmunoprecipitation with Rrp6-TAP or Rrp46-TAP. (A) Western blot validation of TAP-tagged RRP6 and RRP46 used as baits in coimmunoprecipitation assays. The immunoblot shows the bands corresponding to the baits Rrp6-TAP (∼105 kDa) and Rrp46-TAP (∼45 kDa) from the elution fractions, confirming efficient binding to the column. The TAP tag (∼21 kDa) accounts for the shift from native molecular weights (∼84 and ∼25 kDa, respectively). (B) Principal component analysis (PCA) of phosphopeptide intensity profiles. Each point represents one biological replicate of Rrp6 (blue triangles) or Rrp46 (red circles). PC1 and PC2 account for 56.5% and 17.7% of the total variance, respectively. Ellipses represent 95% confidence intervals for each group. Tight clustering of Rrp6 replicates and broader dispersion of Rrp46 samples indicate group-specific phosphoproteomic profiles and differential variance structure.

A total of 737 phosphorylated residues were identified (Supporting Information Table 1), corresponding to phosphosites present on proteins copurifying with the exosome subunits. To refine the phosphoproteomic data set, all identified phosphoproteins were filtered toward rRNA processing and nucleocytoplasmic transport, resulting in 114 candidate phosphosites (Supplementary Table 1). Gene ontology analysis of proteins identified in these affinity-purified fractions showed a prevalence of rRNA processing factors, which was expected due to the essential role played by the exosome in the ribosome maturation pathway (Figure 2). Among the rRNA processing factors recovered, those involved in 5.8S rRNA and SSU maturation were the most abundant (Figure 2), which reinforces the validation of our results since the exosome is directly involved in mature 5.8S formation and associates with the 90S processome. The enriched molecular functions are closely related to rRNA processing activities, indicating that phosphorylation can affect these molecular functions during ribosome assembly.

**Figure 2:**
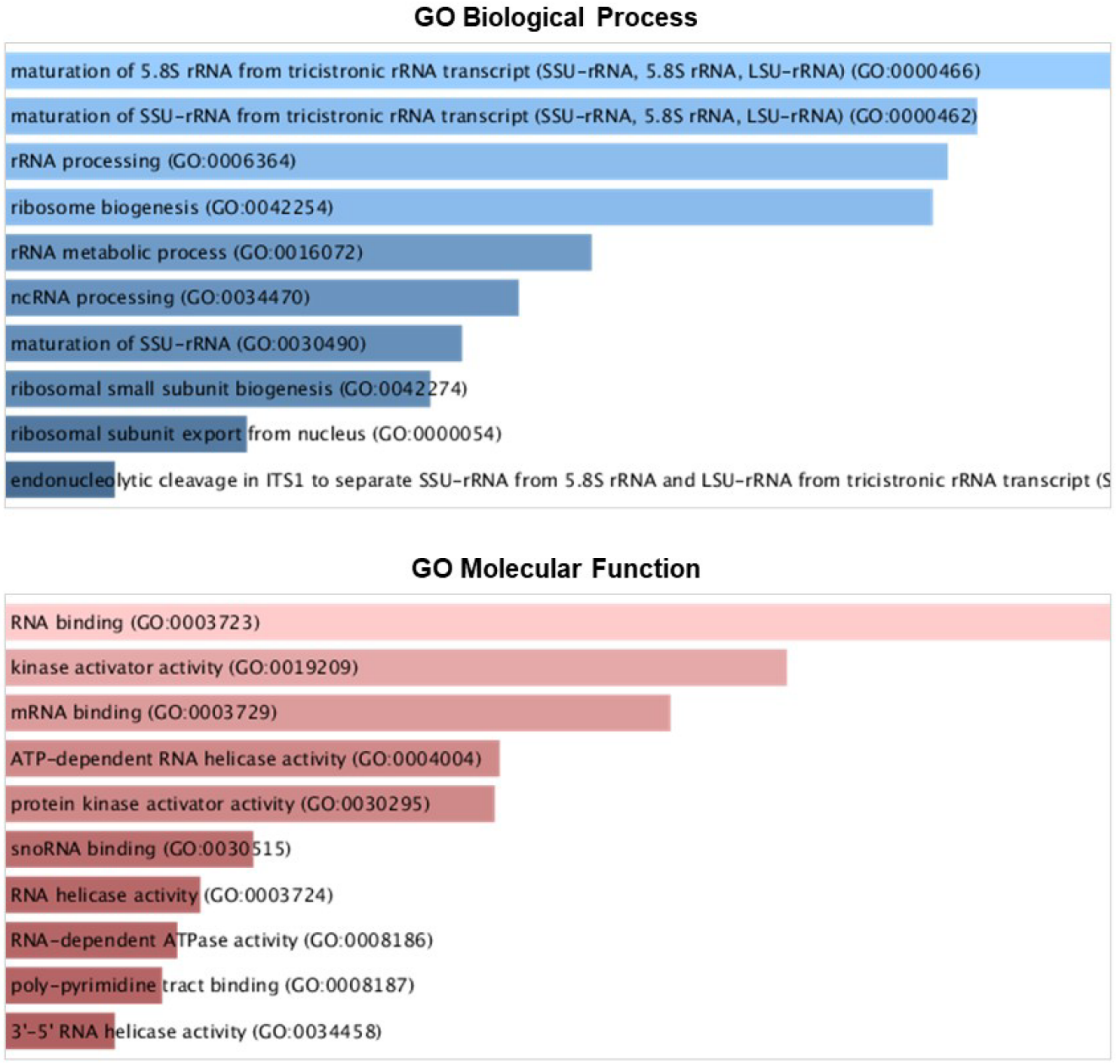
GO terms of biological processes and molecular functions of the proteins copurified with the exosome subunits Rrp6 and Rrp46. (A) Biological process: Ribosome biogenesis factors were the most abundant category in the samples. (B) Molecular function: RNA binding was the prevalent category. Three biological replicates were used in the purifications for the bait proteins were performed.

We investigated the distribution and intensity of phosphorylation events across the two experimental conditions. A heatmap based on phosphosite intensities for rRNA processing-associated proteins and nucleocytoplasmic transport revealed both shared phosphorylation profiles and condition-associated patterns between Rrp46 and Rrp6 (Figure 3).

**Figure 3:**
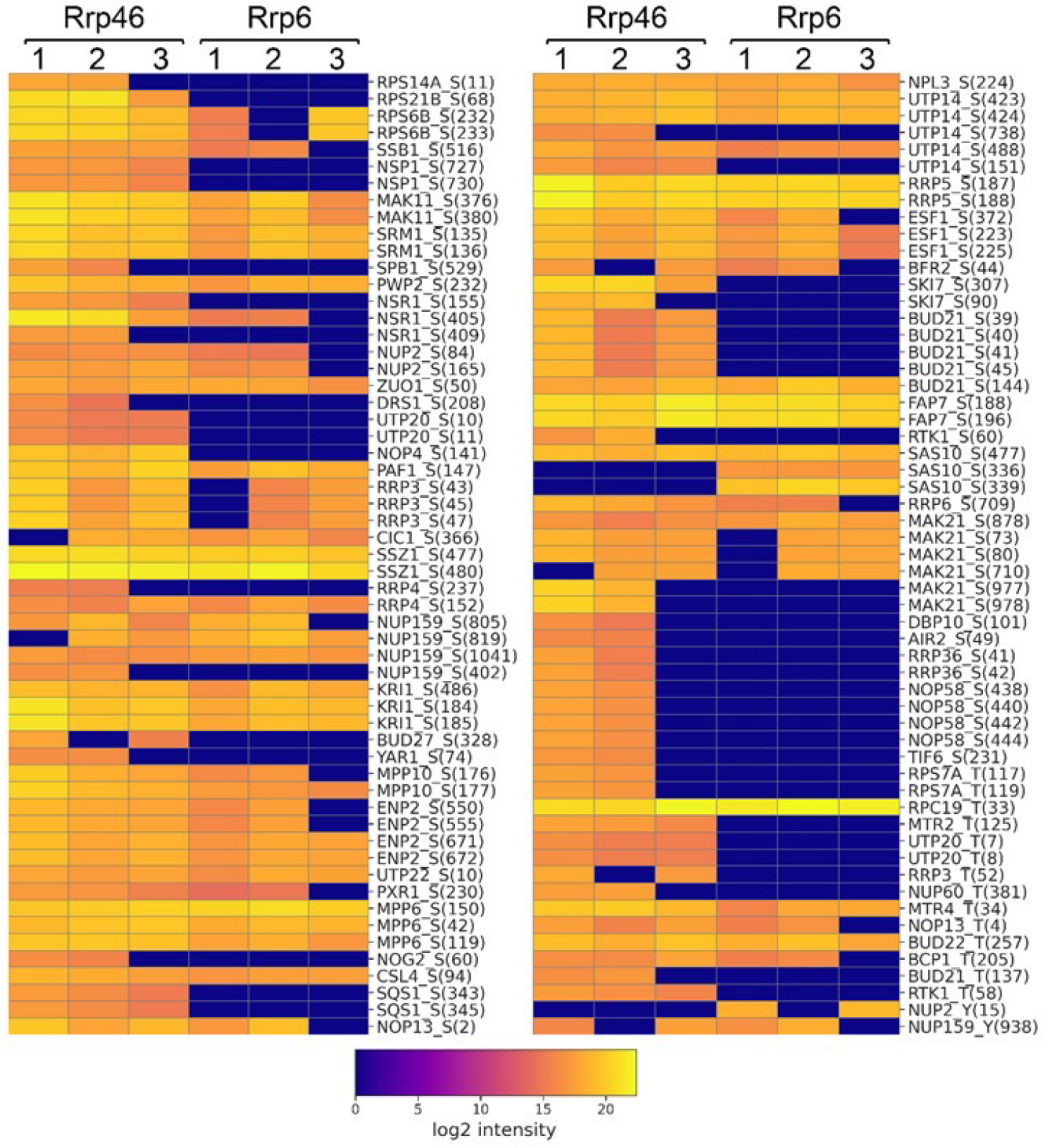
Heatmap of normalized phosphorylation intensities for rRNA processing-associated residues across Rrp6 and Rrp46 immunoprecipitates. Color gradients reflect relative phosphorylation levels across three biological replicates. Distinct clusters represent residues exclusive to Rrp6, Rrp46, or shared between both, highlighting phosphorylation-dependent specificity in exosome-associated preribosomal complexes. Numbers on the top indicate the individual biological replicates used in the co-IP experiments. Numbers in parentheses refer to phosphorylated residue positions.

Utp20, Nop4, Sqs1, and Nog2, among other ribosome maturation factors, were uniquely detected in Rrp46 samples, whereas other assembly factors, including Enp2, Pwp2, Kri1, and Utp22, were phosphorylated in both samples (Figure 3), suggesting a heterogeneity of nuclear exosome complexes. Notably, the phosphosites on the N-terminal unstructured portion of Utp20 detected here have not been previously reported. The selective enrichment of phosphorylated peptides in Rrp46 samples suggests that the association of the exosome at different stages of ribosomal maturation may be mediated by additional assembly factors, other than Utp18 (SSU processome) and Nop53 (pre-60S) via Mtr4-Rrp6 direct interaction.^29^

A smaller set of phosphosites was uniquely detected in the Rrp6 samples. Among these, Sas10 (residues S336 and S339) and the nuclear pore-associated export factor Nup2 (Y15) were consistently phosphorylated only in the Rrp6-associated fractions (Figure 3). Sas10 is functionally linked to the SSU processome and has been shown to bind Rrp6-Rrp47 heterodimer,^30^ confirming the interaction of the exosome with factors involved in early rRNA processing steps in the nucleolus.^8^ Further demonstrating the consistency of our results, proteins with multiple phosphorylation sites showed particular phosphorylation states, depending on the residue and the complex they were purified with. Utp20, for example, had its four phosphorylated residues purified exclusively with Rrp46, while Sas10-S336 and S339 were phosphorylated exclusively in Rrp6 samples. Sas10-S477 and Mpp10, on the other hand, a protein of the same complex as Sas10, were phosphorylated in both samples (Figure 3; Table 1). These differential phosphorylation states might indicate the regulation of protein function, affecting interactions throughout ribosomal maturation.

**Table 1:** Phosphosites in Identified Peptides from Proteins Copurified with Rrp6-TAP or Rrp46-TAP^a^

Complex	Protein	Rrp6 Phosphosites	Rrp46 Phosphosites
Exosome	Csl4	S94	S94
	Rrp4	S152	S152, S237
	Rrp6	S709	S709
Exosome Cofactors	Mpp6	S42, S119, S150	S42, S119, S150
	Mtr4	T34	T34
	Air2	—	S49
	Ski7	—	S90, S307
SSU Processome	Utp14	S423, S424, S488	S151, S423, S424, S488, S738
	Utp20	—	T7, T8, S10, S11
	Utp22	S10	S10
	Sas10	S477	S477
	Sas10	S336, S339	—
	Mpp10	S176, S177	S176, S177
C/D box	Nop58	—	S438, S440, S442, S444
Pre-60S	Tif6	—	S231

a Partial list of proteins identified,highlighting exosome subunits and cofactors, and ribosome maturation factors, with phosphoresidues identified in each protein. Novel posphosites are indicated by blue text in the original figure.

Several phosphosites exhibited comparable phosphorylation levels in both Rrp6 and Rrp46 complexes, suggesting that these modifications play constitutive regulatory roles irrespective of the context of the ribosomal maturation stage with which the exosome is associated. These shared sites were found in central assembly factors such as Mpp10, Utp22, Utp14, Rrp5, Kri1, Enp2, Bud21, Bud22, Esf1, and Pwp2 (Figure 3). These proteins are essential for 90S preribosome formation, and the conserved presence of their phosphorylated forms in both conditions indicates that their regulation is fundamental and that the Exo11 complex remains bound to preribosomal particles at different maturation stages.

In addition to the phosphorylated peptides copurified with the exosome subunits, phosphosites of three different exosome subunits were identified, Csl4-S94, Rrp6-S709, and Rrp4-S152, S237 (Figure 3; Table 1). Interestingly, these residues were phosphorylated in both samples, purified with Rrp6 and Rrp46, with the exception of Rrp4-S237, which was copurified exclusively with Rrp46, suggesting that the phosphorylation of this residue is involved in the cytoplasmic function of the exosome, either for activity control or recruitment of the complex by its cofactors. Importantly, this residue is exposed on the exosome complex structure^31^ (Figure 4; Figure S1), indicating its suitability for being modified. Csl4-S94 and Rrp4-S152 phosphorylation have already been described,^32^ confirming the high reproducibility of our purification method.

**Figure 4:**
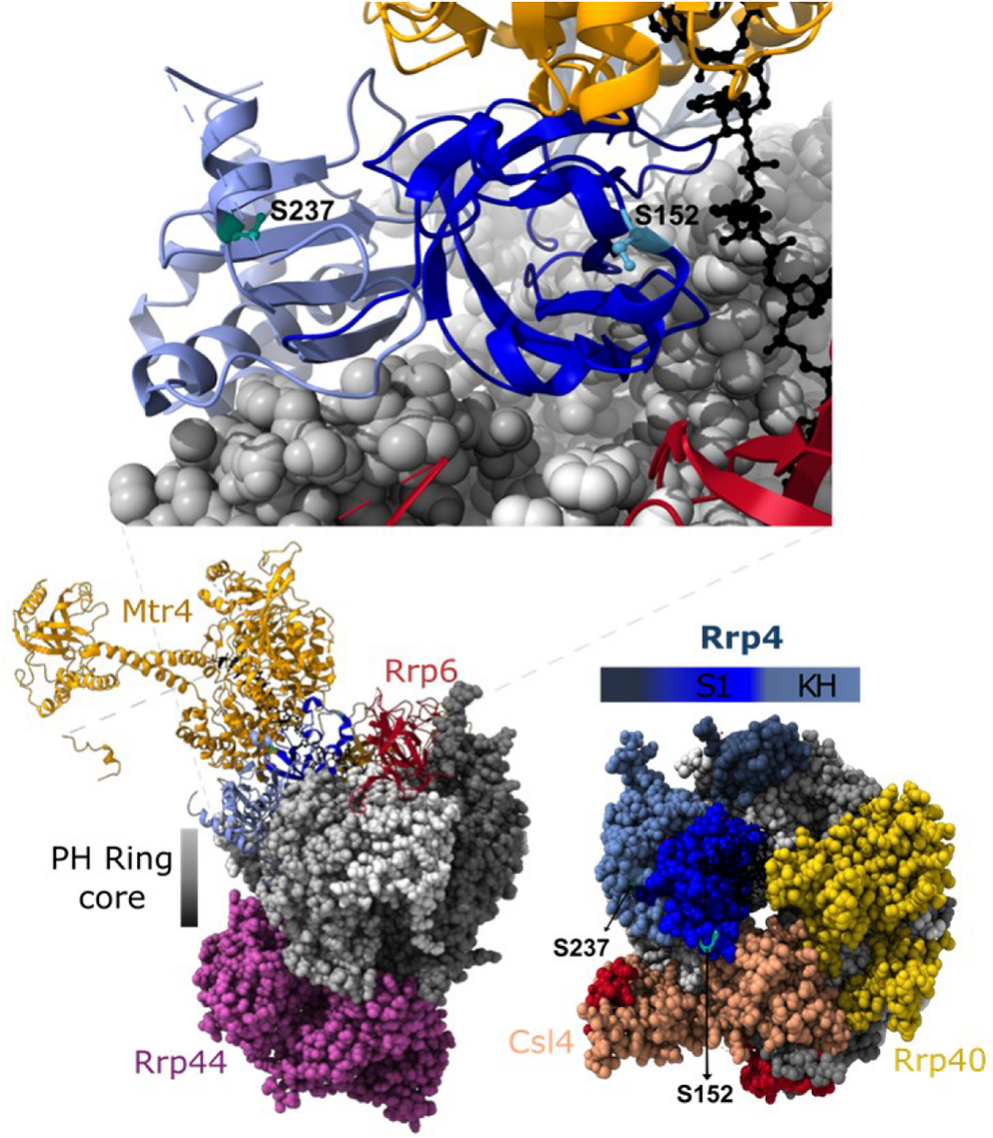
Structure of the exosome, highlighting the positions of Rrp4 phosphorylated residues ser152 and ser237 (indicated in cyan and green, respectively). Structure obtained from PDB 6FSZ.

Csl4-S94 is present in a predicted disordered region of the protein. This phosphosite has been previously reported to be significantly more abundant in the nuclear than in the cytoplasmic exosome, suggesting that phosphorylation at this position may modulate Csl4 RNA-binding dynamics and contribute to compartment-specific regulation of exosome activity.^33^ Rrp4-S152, on the other hand, is embedded in its S1 RNA-binding domain and is accessible for post-translational modifications (Figure 4; Figure S1) and is conserved in the human ortholog EXOSC2.^32,34^ The importance of Ser152 phosphorylation for Rrp4 function was assessed by substituting Ala for this Ser (Rrp4-S152A) and analyzing the growth of yeast cells expressing this mutant. For this analysis, the endogenous promoter of *RRP4* was replaced by *GAL1* promoter, which is activated in the presence of galactose and inhibited in glucose medium, and this strain (*GAL1:Myc-RRP4*) was transformed with a plasmid expressing either wild-type Rrp4 or the mutant Rrp4-S152A, fused to the HA tag and under the control of the constitutive promoter *ADH1*. Growth was then analyzed in glucose medium under conditions in which only the plasmid-encoded Rrp4-HA proteins are expressed. Despite the evolutionary conservation of this residue, however, its change to the nonphosphorylatable Ala does not affect growth (Figure 5), suggesting that phosphorylation at position 152 does not interfere with the RNA-binding activity of Rrp4.

**Figure 5:**
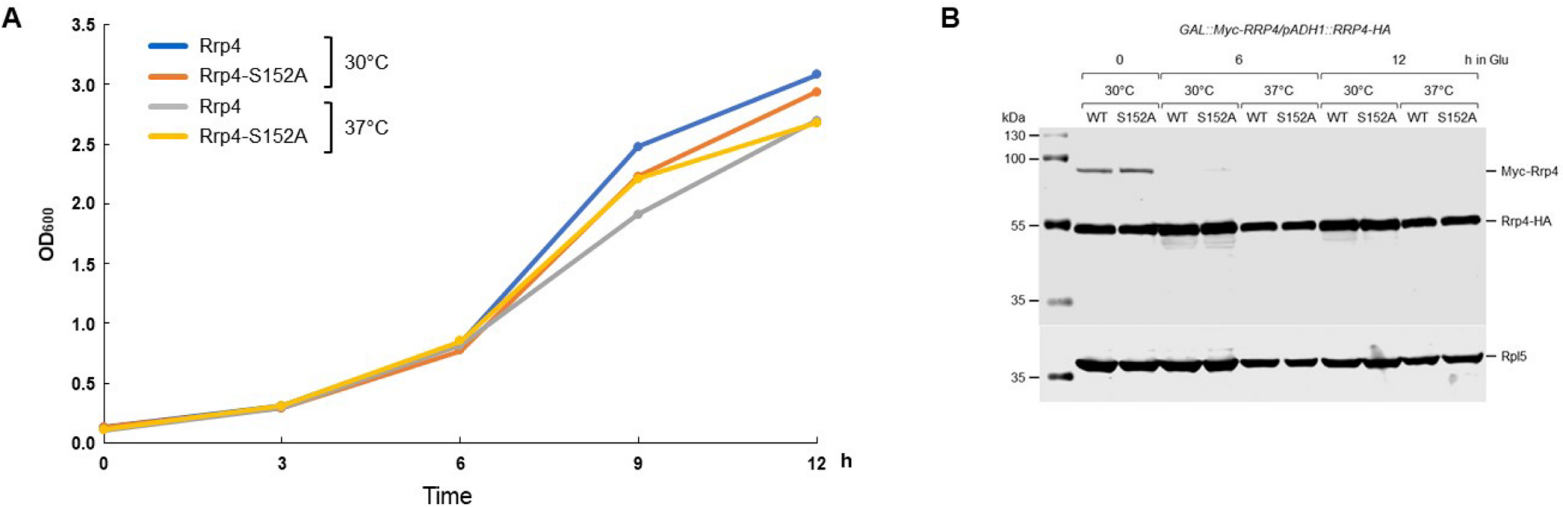
Effects of Rrp4-S152A mutation on cell growth. Plasmid-borne wild-type Rrp4-HA or mutant S152A were expressed in *GAL:RRP4-Myc* cells, and growth was monitored over time in glucose medium, a condition in which only the plasmid copies of Rrp4 are expressed. (A) Growth curve in glucose medium. (B) Western blot for detection of chromosomal-encoded Myc-Rrp4 and plasmid-encoded WT and mutant (S152A) Rrp4 fused to HA tag. Endogenous Rpl5 was used as a loading control.

Rrp6-S709 is present in the lasso domain, involved in RNA binding and stimulation of exosome activity, and is inserted in the canonical nuclear localization signal (NLS) of Rrp6 (697–723),^35,36^ potentially affecting both RNA binding and Rrp6 interaction with karyopherins. Phosphosites S42, S119, and S150 of the exosome cofactor Mpp6 ^37,38^ were found in both samples (Table 1). Mpp6 has already been identified as a phosphorylated protein in high-throughput analyses,^39^ validating our results. A minimal portion of Mpp6 comprising amino acid positions 90 through 118 was crystallized in complex with the exosome and shown to bind directly Rrp40.^38^ Because of its proximity to the Mpp6 portion binding the exosome, it is possible, therefore, that phosphorylation of S119 might influence the Mpp6 association with the exosome, possibly positively, since this phosphosite was recovered with both baits.

Mtr4 has been shown to participate in pre-rRNA maturation by directly interacting and recruiting the exosome for processing.^40,41^ Although the phosphosite T34 was detected here and also previously,^39^ Mtr4 N-terminal 75 amino acids have been shown not to be necessary for the interaction with the exosome.^41^ T34 phosphorylation may therefore not be involved in exosome activity control. Mtr4 is a subunit of the TRAMP complex, of which Air2 and Trf4 are also part.^15^ We identified the phosphosite S49 of Air2, in a predicted low-complexity region of the protein that controls Mtr4 RNA-binding activity.^42^ S49 does not directly interact with Mtr4 (distances >20 Å), but the intrinsically disordered region that contains S49 is enriched in charged residues that promote its high flexibility, suggesting that phosphorylation of S49 might reorient it toward Mtr4. This could stabilize the Air2-Mtr4 interaction and modulate TRAMP complex activity during rRNA processing (Figure S2). Interestingly, although the TRAMP complex localizes to the nucleus, Air2-S49 was only found phosphorylated in the Rrp46 sample, confirming that by using this bait, we isolate both cytoplasmic and nuclear exosome complexes. These results also further imply a heterogeneity of nuclear exosome complexes, which might have distinct functional roles.

Phosphorylated Ski7, a known cytoplasmic exosome adaptor,^18^ was exclusively detected in Rrp46 samples (Figure 3; Table 1), validating the use of this core subunit as bait for identifying exosome interactors. Phosphosite S90 is present in the middle of the N-terminal portion of Ski7, between the SKI complex-interacting and the exosome-interacting regions of Ski7, whereas S307 is inserted in its GTPase domain (Figure 6; Figure S3), which suggests that the phosphorylation of these residues might affect Ski7 function.^43,44^

**Figure 6:**
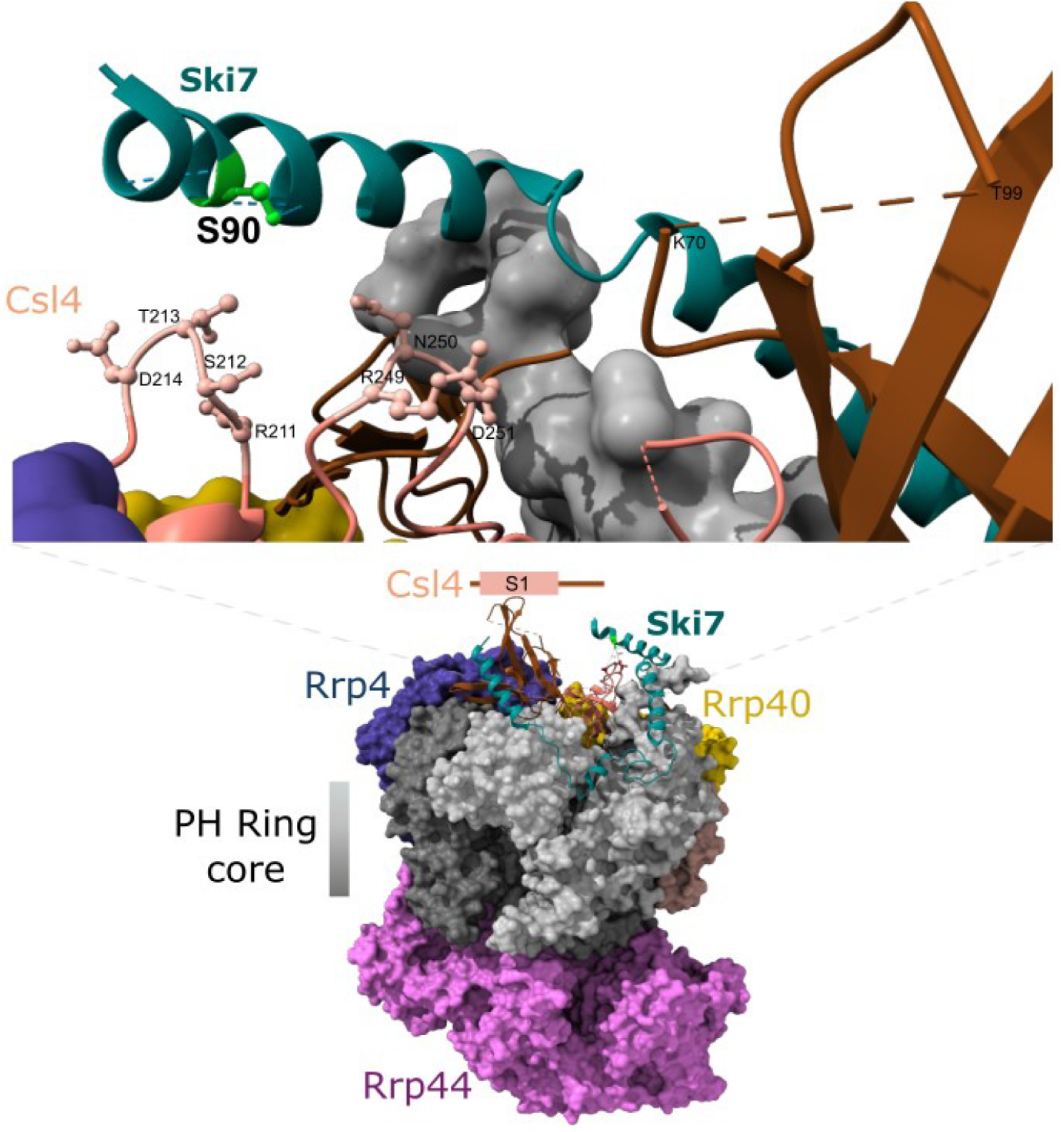
Structure of Ski7 bound to the exosome, highlighting the position of Ski7 phosphorylated residue Ser90 (light green). Structure obtained from PDB 5GO6.

Based on the observation that overexpression of Ski7 traps the exosome in the cytoplasm, therefore affecting ribosome synthesis,^27^ to confirm the hypothesis that S90 phosphorylation might have a functional relevance, the genes of wild-type Ski7 and the mutant Ski7-S90A were cloned into a plasmid under the control of *MET25* promoter and transformed into *Δski7* strain to test the effects of their overexpression on cell growth. The results show that while Ski7 overexpression strongly inhibits growth, the nonphosphorylatable Ski7-S90A does not impair growth (Figure 7), growing very similarly to the condition when Ski7 is expressed at low levels (+Met; Figure 7). These results confirm the importance of Ski7 phosphorylation for the regulation of its function.

**Figure 7:**
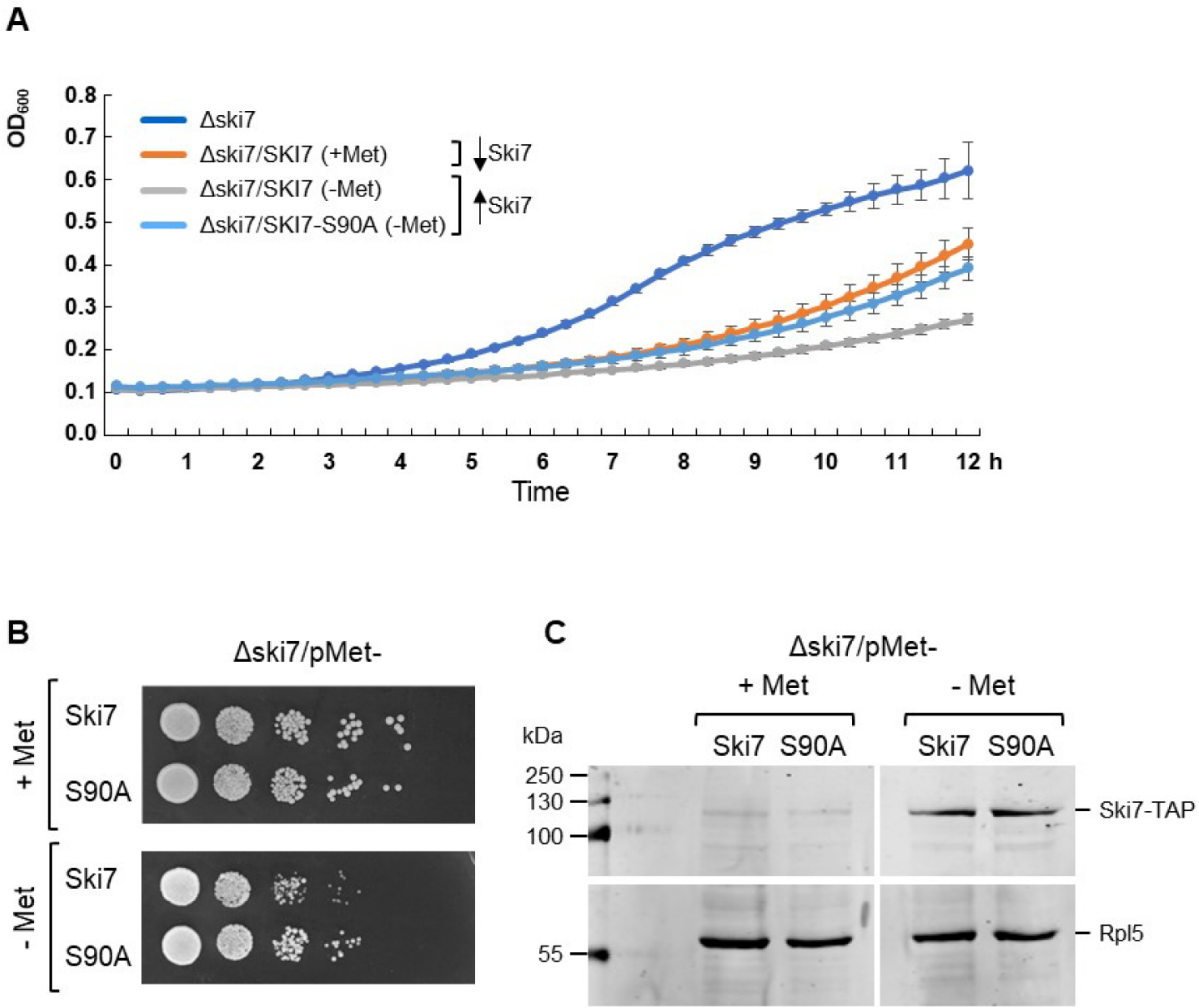
Effects of overexpression of Ski7 on cell growth. Plasmid-borne wild-type Ski7 or mutant Ski7-S90A was overexpressed in Δski7 cells, and growth was monitored over time. + Met: repression of Ski7 expression; -Met induction of Ski7 expression. (A) Growth curve in liquid medium. (B) Serial dilution of cells in OD 0.1 on plates with solid medium. (C) Western blot for detection of plasmid-encoded WT and mutant (S90A) Ski7 fused to TAP tag, whose expression is activated in the absence of Met in the medium. Rpl5 was used as a loading control.

SSU processome subunit Utp14 phosphosites were also recovered in our samples, with the phosphoresidues S423, S424, and S488 identified in both Rrp6 and Rrp46 eluates (Table 1). These residues are present in the Utp14 portion involved in the recruitment of the DEAH-box helicase Dhr1,^45^ and, therefore, the phosphorylation of these Ser residues might have functional relevance. Interestingly, we also identified phospho-S738 exclusively in Rrp6 samples. This residue is located in the Utp14 region responsible for Dhr1 activation, which occurs right after the release of Rrp6 from the SSU processome.^45^ Importantly, all of the Utp14 phosphosites have previously been identified in global yeast proteomics analyses,^39^ validating our results.

To investigate the evolutionary relevance of the phosphorylation sites identified here, we assessed the conservation of phosphorylated residues across orthologous proteins in humans and mice. The sequence alignments identified conserved phosphorylation sites in the corresponding positions of the orthologous sequences. The majority of phosphosites (approximately 58%) were not conserved in either human or mouse orthologues, but a subset of residues (12.4%) was conserved in both mammalian species, including those of Air2 (S49), Ctr9 (S1015, 1017), Enp2 (S550, S555), Nop13 (S2), Pxr1 (S230), Rps21B (S68), Rps6B (S232, S233), Rps7A (T117, T119), Rrp3 (S43, S45, S47), and Rrp4 (S152) (Table 2). The observed conservation of these phosphoresidues suggests a potential functional importance across eukaryotes.

**Table 2:** Identified Phosphosites Conserved in Human and Mouse^a^

Protein Name	Peptide Sequence	Peptide Position	Phosphorylated Residue Conserved in Human and Mouse
Air2	YFGVSDDDDKDA	45–55	Ser49
CTR9	AKKTLSDSDED	1010–1020	Ser1015–Ser1017
ENP2	YEDEESDEEES	545–555	Ser550–Ser555
NOP13	MSETELSKED	1–10	Ser2
NRD1	RNRSRSPPAPF	260–270	Ser265
PAF1	RRLDDGDSDDEN	140–150	Ser147
PXR1	LKSSESASNIP	225–235	Ser230
RPS21B	RSRGESDDSLN	60–70	Ser68
RPS6B	IRKRRASSLKA	226–236	Ser232–Ser233
RPS7A	SRTLTAVHDKI	115–125	Thr117–Thr119
RRP3	EGGSESDSEED	40–50	Ser43–Ser45–Ser47
RRP4	RKSESDELQMR	150–160	Ser152
SBB1	AVGRLSSEEIEK	510–520	Ser516

a Partial list of proteins identified, highlighting exosome cofactors, ribosome assembly factors, and ribosomal proteins. Phosphoresidues in each protein are indicated.

To evaluate the functional importance of the identified phosphosites, we performed a broad evolutionary analysis focusing on ribosomal proteins and ribosome assembly factors. We aligned the phosphopeptides identified for these proteins with their corresponding sequences from 16 additional organisms and observed that many of these conserved peptides also contain conserved phosphoresidues. We then analyzed the relative proportions of serine, threonine, and tyrosine (STY) residues, as well as aspartic acid and glutamic acid (DE), relative to the total conserved peptides (Figure 8).

**Figure 8:**
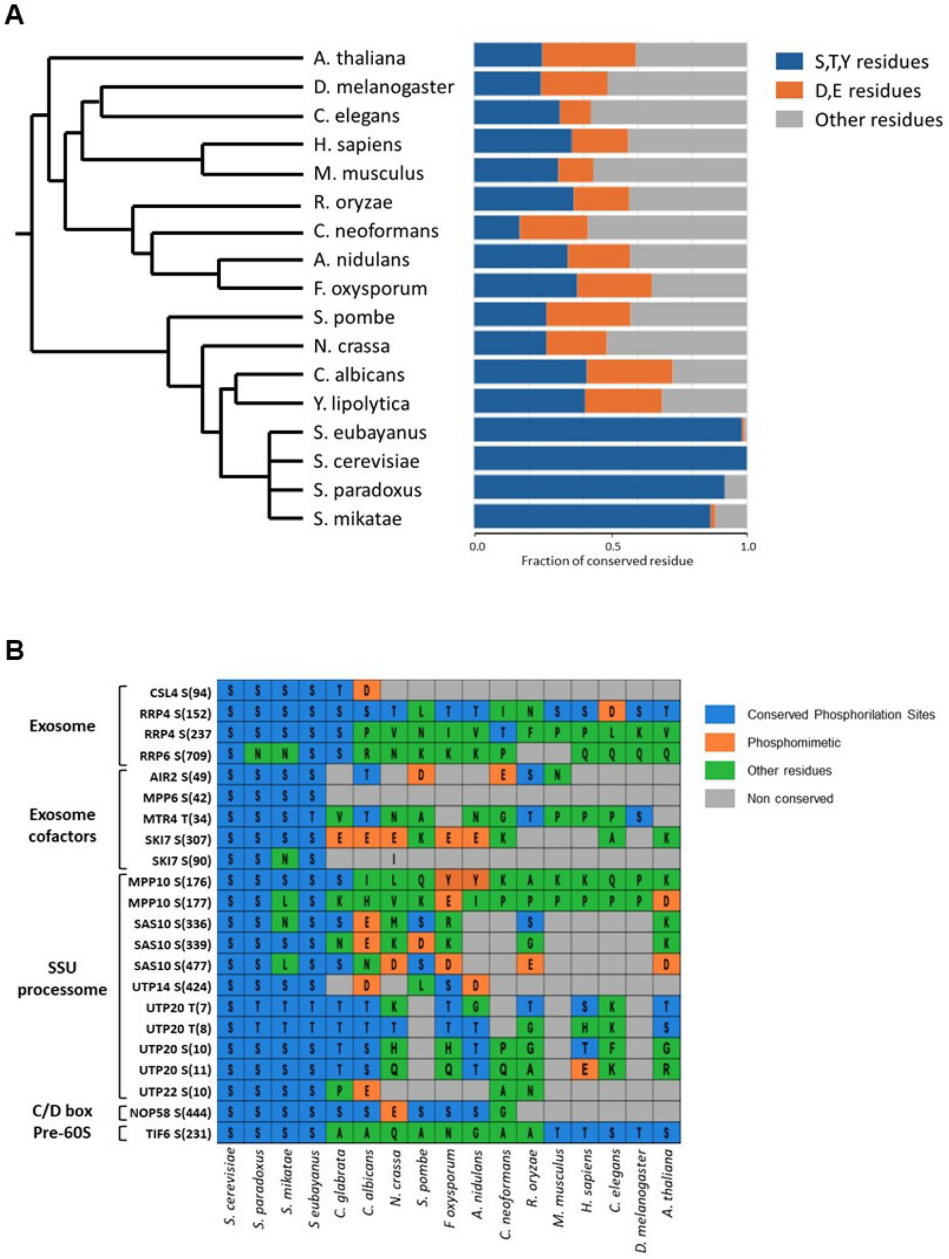
Analysis of phospho peptides conservation. (A) Phylogenetic tree showing the percentage of conserved ribosome residues. The tree was constructed using 16 organisms in addition to S. *cerevisiae*, and the clades were generated using the iTOL (Interactive Tree of Life, https://itol.embl.de/itol.cgi) program. Ribosomal associated residues identified in our phosphoproteome analysis were used to generate the alignment and to calculate the relative fraction of conserved residues to each species. (B) Plot showing the conservation of phosphoresidues in ribosomal proteins or assembly factors. Ribosomal associated residues identified in our phosphoproteome analysis were used to generate the alignment and to calculate the relative fraction of conserved residues to each species. The analyzed residues were grouped by complex and colored according to their phosphorylation-related characteristics: Blue: S, T, Y residues with conserved serine, threonine, or tyrosine. Orange: Conserved residues in which STY is replaced by aspartic acid or glutamic acid (“D”, “E”). Green: Other residues different from STY or DE. Gray: Residues that are not conserved.

The *Saccharomycetaceae* clade has a strong conservation of STY-containing peptides (Figure 8A, blue bars). As expected, the degree of conservation decreases as more evolutionarily distant the organisms are from *S. cerevisiae*, as indicated by an increase in residues unrelated to phosphorylation (gray bars). In more distant organisms, the conservation of phosphorylatable “STY” residues decreases to approximately 25%. Interestingly, this reduction is accompanied by an increase in phosphomimetic (negatively charged) amino acids (D or E), represented by orange bars. Although these organisms have lost their phosphorylatable residues at these positions, their replacement by phosphomimetic residues suggests that the negative charge at these sites is important for protein structure or function (Figure 8A).

The identified *S. cerevisiae* phosphoresidues of exosome subunits and cofactors, and of ribosome assembly factors were aligned with sequences from 16 additional organisms and grouped by complex to analyze the evolutionary conservation of the phosphorylation. The conservation profile mirrors the one presented above, showing the phosphorylatable S, T, and Y residues conserved positions in *Saccharomycetaceae*, while D and E substitute these residues in more distant organisms, preserving the negative charge (Figure 8B). Interestingly, several residues are conserved from yeast to higher eukaryotes. This conservation underscores the importance of these phosphorylation sites for the structure and function of the RNA exosome complex and ribosome assembly factors, highlighting the role of phosphorylation as a regulatory mechanism in RNA metabolism.

## Discussion

The RNA exosome is involved in the processing and degradation of all classes of RNA, both in nucleus and cytoplasm.^3,46–48^ In each cellular compartment, the exosome interacts with and is regulated by a specific set of cofactors and interactors,^2^ and these protein interactions may be affected by phosphorylation. In this study, we present a phosphoproteomic investigation of proteins associated with the *Saccharomyces cerevisiae* exosome by using subunits Rrp6 and Rrp46 as baits, and show evidence that phosphorylation may contribute to the structural and regulatory dynamics of the exosome in association with distinct preribosomal intermediate particles and cytoplasmic cofactors.

The majority of proteins interacting with the exosome participate in the RNA processing pathway, and our data show that these factors exhibit a large number of phosphorylated residues, with a predominance of serine phosphorylation, consistent with global eukaryotic phosphoproteome patterns.^49^ Phosphorylation was detected both within structural domains and in intrinsically disordered regions (IDRs), with the latter being known for facilitating dynamic protein interactions. Our findings are in agreement with previous studies demonstrating that the recruitment of the exosome to preribosomes is mediated by cofactors^3,50^ and show their phosphorylation status in distinct complexes, which may influence these interactions.

Although most of the phosphosites isolated here have already been identified in global analyses,^39^ which further validates our data, we detected phosphoresidues being copurified with the exosome as part of preribosomal particles, showing the maturation stage in which their phosphorylation is functionally relevant. In addition, we also detected phosphosites that were not previously described. The evidence that the exosome is associated with preribosomes is given by the number of proteins identified that participate in various preribosomal particle steps. We identified proteins associated with the preribosomal particle 90S (e.g., Utp14, Mpp10), pre-60S (e.g., Nop4, Tif6), proteins involved in nuclear transport of ribosomal particles (e.g., Mtr2, Srm1), subunits of snoRNP particles (e.g., Nop58), ribosomal proteins (e.g., Rps7, Rps14, P1A/B, P2), and nuclear pore complex components (e.g., Nup2, Nup159), which give evidence of the participation of the exosome in multiple steps of ribosomal maturation. The distinct phosphosites identified in the two samples further strengthened this conclusion. On the basis of these differences, we also hypothesize that the exosome complexes present in the nucleus may not be homogeneous but rather exist as Exo11 (containing Rrp6) or Exo10 (without Rrp6). This conclusion could also be inferred by previous coimmunoprecipitation of the exosome with Rrp43, in which Rrp6 was not copurified,^26^ suggesting a loose association of this subunit with the exosome core.

In addition to ribosome factors, we identified the phosphorylated exosome subunits Csl4, Rrp4, and Rrp6. Interestingly, the Rrp4-S237 phosphosite was only identified in Rrp46 samples, strengthening the hypothesis of these phosphorylation events being important for function, in this case, probably in the cytoplasm or as part of the nuclear Exo10. Furthermore, the cytoplasmic exosome cofactor Ski7 was identified exclusively in Rrp46 samples, validating the purification method. The importance of Ski7-S90 phosphorylation was confirmed here by changing this residue to alanine, which abolishes the negative effect of Ski7 overexpression on cell growth. Interestingly, S90 is part of helix H3 in the SKI complex-interacting portion of Ski7, in close proximity to the exosome-interacting region of Ski7 (positions 116–225; Figure 6).^44,51^ It is possible, therefore, that phosphorylation of S90 might influence Ski7 interaction with the SKI complex and also affect its interaction with the exosome, since Ski7 overexpression has been shown to trap the exosome in the cytoplasm.^27^ Our results corroborate the hypothesis that S90 phosphorylation is important for function and might modulate cytoplasmic RNA degradation pathways.

The analysis of the phosphorylated residues across 17 organisms ranging from yeast to humans shows their evolutionary conservation, further strengthening the validity of our results. Also confirming the importance of the phosphorylation sites identified here, posttranslational modifications of *S. pombe* exosome subunits Dis3 and Rrp6, and cofactor Mtr4, have been shown to be essential for normal cell growth and to fine-tune exosome activity regulation.^25^

In conclusion, here we have identified ribosome assembly factors that are phosphorylated, a modification that may affect their affinity for binding the intermediate preribosomal particles, and show evidence that the exosome may not be a homogeneous complex in the nucleus, but rather be present as Exo11 and Exo10 + Rrp6 in this cell compartment, and its subunits may also be subject to phosphorylation for functional regulation.

## Supplementary Material





## Data Availability

The mass spectrometry proteomics data have been deposited in the PRIDE Archive (http://www.ebi.ac.uk/pride/archive/) via the PRIDE partner repository. The data set identifier is PXD073587.

## Notes

Figure S1: Analysis of solvent-exposed RNA exosome surface. Figure S2: Implication of the Air2 phosphorylation site in the Air2-Mtr4 complex structure. Figure S3: Positioning of Ski7 phosphorylation site when associated with the exosome (PDF)
Table S1: List of exosome-associated phosphopeptides (XLSX)

## #

Laboratory of Chemistry and Function of Proteins and Peptides, Center for Biosciences and Biotechnology, State University of Northern Fluminense Darcy Ribeiro, Camposdos Goytacazes, Rio de Janeiro 28013–600, Brazil
