# Supplementary material for: Phosphorylation Patterns of Preribosomal Proteins Associated with the RNA Exosome
Source: J Proteome Res. 2026 Jun 29;25(8):4050–61. doi: 10.1021/acs.jproteome.6c00095 (PMC13459559; doi:10.1021/acs.jproteome.6c00095)
Supplement: Supplementary file 1 [file pr6c00095_si_001.pdf]

Supporting information

Phosphorylation patterns of pre-ribosomal proteins associated with the RNA exosome

Felipe A. Almeida[1,2], Valdir Gomes Neto[1], Rebeca B. Jantsch[1], Moises R. A. Barros[1], L. Paola P. Cepeda[1], Bruno R. S. Queiroz[1], Anna B. Machado[1], Ana P.J. Menezes[3], Julia P. C. Da Cunha[3] and Carla C. Oliveira[1]*

[1]Department of Biochemistry, Institute of Chemistry, University of São Paulo, 05508-000, São Paulo, SP, Brazil.

[2]Current address: Laboratory of Chemistry and Function of Proteins and Peptides, Center for Biosciences and Biotechnology, State University of Northern Fluminense Darcy Ribeiro, 28013– 600, Campos dos Goytacazes, RJ, Brazil

[3]Cell Cycle Laboratory, Center of Toxins, Immune Response and Cell Signaling-Center for Research on Toxins, Immune-response, and Cell Signaling (CeTICS), Butantan Institute, São Paulo 05503-900 SP, Brazil.

* Corresponding author.

Figure S1 – Analysis of solvent-exposed RNA exosome surface.

Figure S2 – Implication of the Air2 phosphorylation site in the Air2-Mtr4 complex structure.

Figure S3 – Positioning of Ski7 phosphorylation site when associated with the exosome.

Table S1 – List of exosome-associated phosphopeptides.

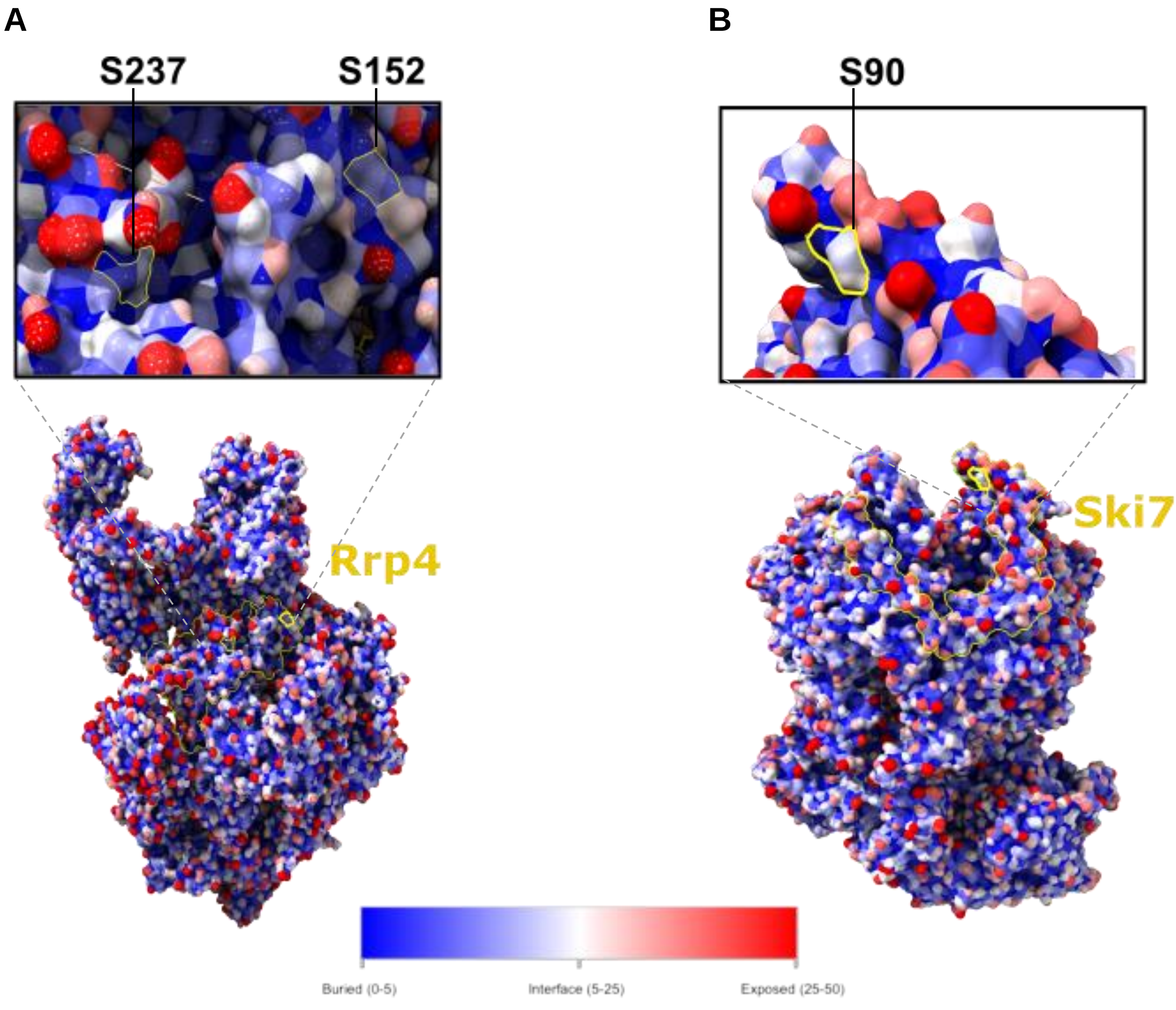

Figure S1. Analysis of the solvent-accessible surface area (SASA) of the *Saccharomyces cerevisiae* exosome complex. The molecular surface of the *S. cerevisiae* exosome was generated to analyze specific residues identified in this phosphoproteomics study. (**A**) S152 and S237 of Rrp4 (PDB 6FSZ). S152 is located at an accessible interface, while S237 is buried. (**B**) S90 of Ski7 is exposed on the surface (PDB 5G06).

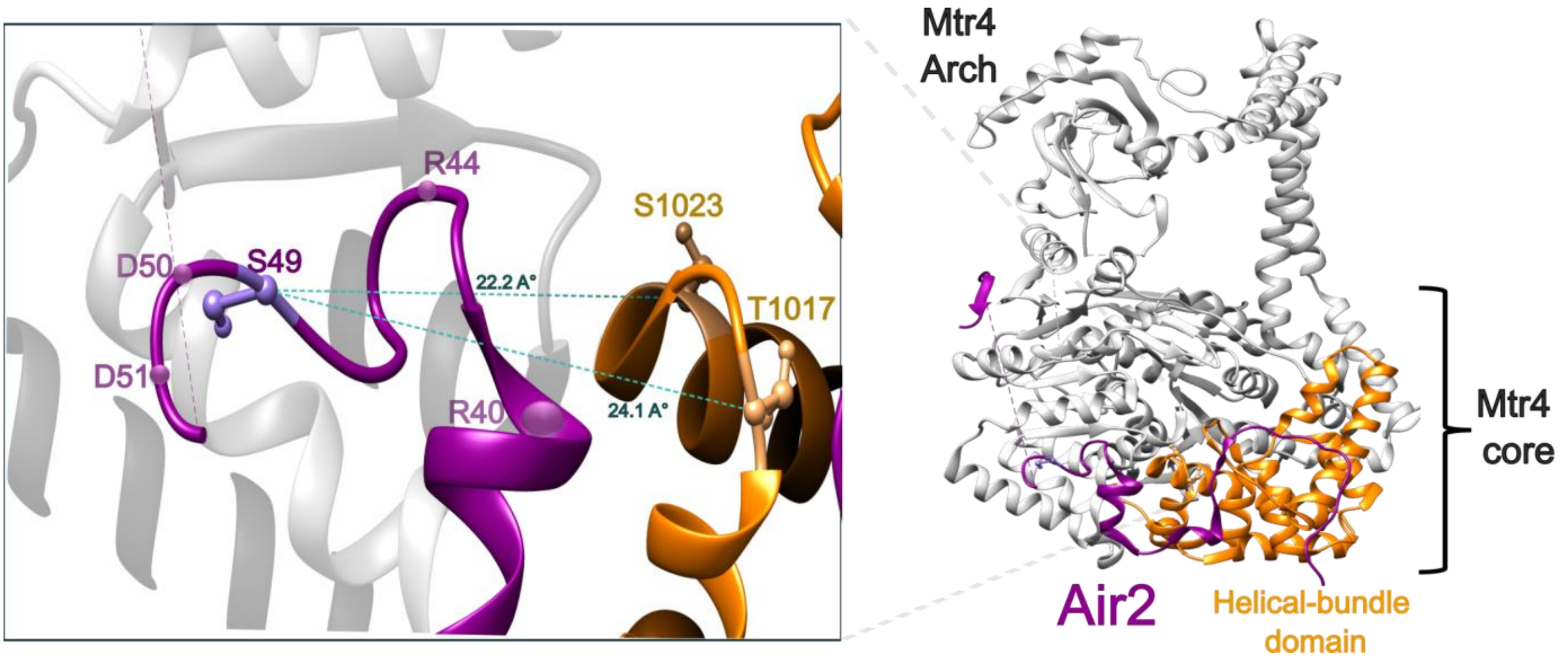

Figure S2. The phosphorylation site of the Mtr4-Air2 complex. A diagram illustrating the TRAMP complex of *S. cerevisiae* (PDB 4UCU) shows Mtr4 (gray and orange), highlighting the phosphorylation sites. Air2-S49 (violet) was identified as phosphorylated in this study. It is located in a flexible region flanked by charged residues (R40, R44, D50, D51). Its phosphorylation might activate a conformational switch that establishes electrostatic contacts with the Mtr4 helical bundle domain, coupling the phosphorylation signal to activity in the TRAMP complex during rRNA processing.

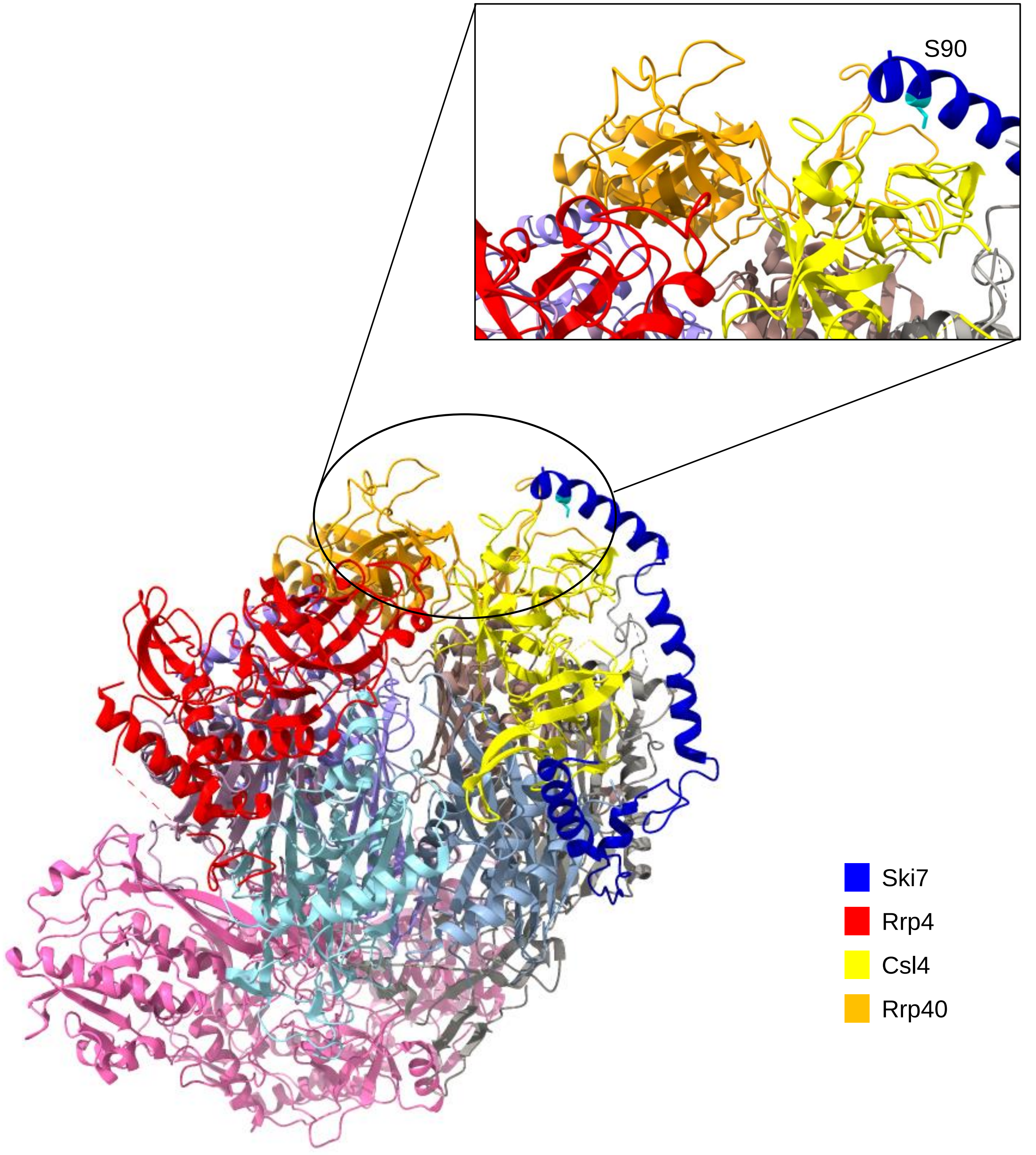

Figure S3. **Ribbon representation of the structure of Ski7 bound to the exosome**. The Ski7 phosphorylated residue Ser90 is highlighted in cyan. Structure obtained from PDB 5GO6.
